# Recent insights into bonding technologies in restructured meat production: A review

**DOI:** 10.1016/j.fochx.2024.101712

**Published:** 2024-08-05

**Authors:** Zongyao Ren, Zhijie Li, Zhonghai Hu, Wenyun Xia, Mi Zhou, Zhenjie Pan, Jingjun Li, Zongyuan Zhen

**Affiliations:** a College of Food Engineering, Anhui Science and Technology University, Chuzhou 233100, China; b Lu'an Longxiang Gourmet Poultry Co., Ltd., Lu'an 237400, China; c Food and Drug Inspection and Testing Center, Neijiang 641000, China; d Anhui Liuxiangge Food Co., Ltd., Chuzhou 239000, China; e Associated Discipline Key Laboratory of Whole Grain Nutrition and High-Value Utilization, Chuzhou 233100, China; f Anhui Provincial Key Laboratory of Functional Agriculture and Functional Foods, Chuzhou 233100, China

**Keywords:** Glucono-delta-lactone, Fibrin sealant, Gel emulsification, Transglutaminase, Gelatin

## Abstract

Restructuring meat products is one way of improving material utilization and economic efficiency. In this process of combining meat pieces or granules to form larger pieces of meat, the additives and processing techniques employed in bonding the restructured meat play crucial roles in the formation of the structure and appearance of the meat while simultaneously reducing nutrient and water loss and enhancing flavor. This study reviews the adhesives commonly used in meat recombination technology, including transglutaminase, glucono-delta-lactone, fibrin, gelatin, and gel emulsifiers such as hydrophilic colloid, phosphate, starch, and cellulose. Additionally, processing technologies such as high-pressure, ultrasonic, vacuum-assisted, microwave, and three-dimensional printing are discussed, with emphasis on their principles, properties, functionalities, and safety. The study further summarizes the application and research progress of various bonding techniques in restructured meat. It analyzes the advantages, challenges, and development prospects of these techniques to provide support for further research in this field.

## Highlights

• Progress in bonding technology and additive-processing synergy in restructured meat.
• Evaluation of the effects of TG, GDL, FS, gelatin, emulsifiers in meat restructuring.
• Assessment of safety and functionality of various bonding techniques.

## Graphical abstract

**Figure:**
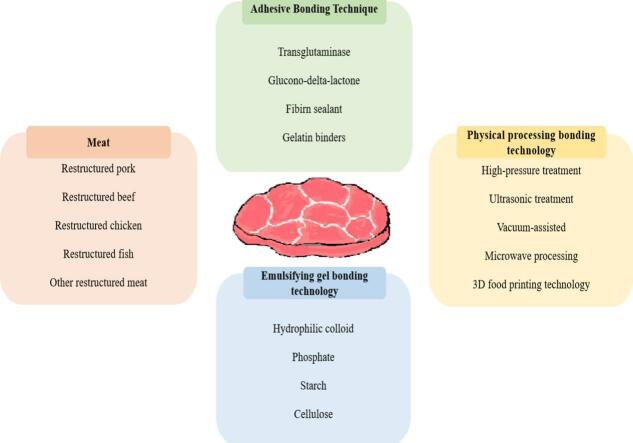

## 1 Introduction

Meat is an essential part of people's daily diet. The rapid growth in population and improvement in living standards have led to a significant increase in the demand for meat products. This surge in demand has prompted the advancement of meat processing towards industrialization. However, within the industrial production of meat products, a large number of meat pieces, minced meat, meat granules, and other by-products often remain underutilized (Thakur et al., 2023). The emergence of restructured meat technology in the 1960s addressed this inefficiency by using additives and the adhesive effect of the processing technology to change the original tissue structure of the meat. This transformation enabled the muscle and fat tissues to be rationally distributed to achieve functional properties similar to those of conventional meat sold as fresh meat after freezing or as cooked meat products after preheating treatment (Liu et al., 2017). Currently, restructured meat has become a consumer-recognized product type that accounts for a significant portion of fresh meat ingredients, particularly in catering industry (Zeng et al., 2019). Although research on restructured meat started late in China, outcomes from related studies have been applied in industrial production, such as restructured beef (Li et al., 2021), restructured steak (Park et al., 2023), restructured pork (Tseng et al., 2006), and restructured pork chops (Nachomkamon et al., 2022). Adhesive and bonding technology plays a crucial role in the process of restructured meat, enhancing not only the product's ideal organizational structure and taste but also its tenderness, water retention, flavor, and quality. This study reviews the principle, property, functionality, and safety of the typical bonding technology used in the processing of restructured meat. Furthermore, it provides a summary and analysis of their application and research progress to offer insights for further exploration of restructured meat processing technology.

## 2 Classification and principles of meat restructuring techniques

**Fig. 1:**
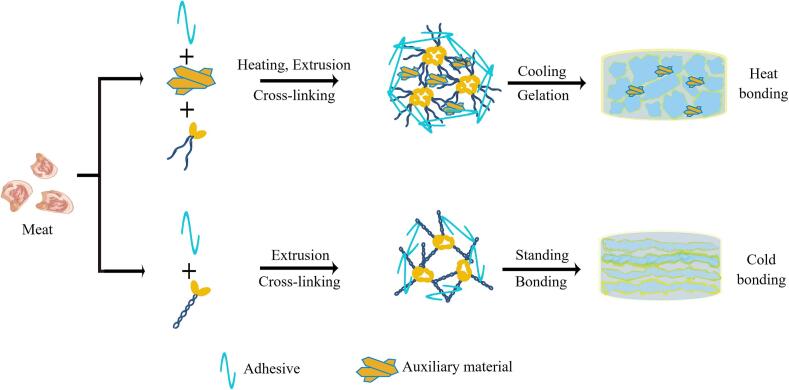
Mechanism of restructured meat bonding process (By Figdraw).

The restructuring of meat involves two main processes: hot and cold bonding (Fig. 1). Starch, hydrophilic colloids, glucono delta-lactone, and gelatin are classified as thermal adhesives, whereas fibrin sealant fall under cold adhesives. TGase is suitable for both cold and thermal bonding (Fu et al., 2018; Zhou et al., 2017). The cold bonding method, which does not involve heating, primarily utilizes adhesives, emulsifying gels, and mechanical external forces to recombine the ground meat, imparting unique texture and bonding characteristics to the product. Common meat products made via cold bonding processing include restructured steak and mutton rolls. These products exhibit relatively low elasticity and closely resemble the flavor profile of raw meat, and they often require to be frozen for storage. On the other hand, hot bonding involves the use of myofibrillar protein or the addition of fat, starch, hydrophilic colloid, and non-meat protein by heating the ground meat or meat particles to form a multi-component heat-induced gel (Bai et al., 2023). This gel is primarily composed of the cross-network structure formed by the denatured myosin or actomyosin (Zhang et al., 2024). Common meat products made of hot bonding processing include restructured ham and emulsified sausage. After heat treatment, these meat products exhibit enhanced compactness, superior elasticity, and richer flavor due to the addition of auxiliary materials. Both bonding processes yield a heat-stable gel that ensures the product is firm and intact during heat processing.

## 3 Bonding techniques in restructured meat

The bonding technology employed in restructured meat can be categorized into three main types based on their process mechanisms: adhesive bonding technology, gel emulsion bonding technology, and physical processing bonding technology. These bonding techniques utilize different adhesives, gel emulsifiers, or processes to achieve their objectives.

### 3.1 Adhesive bonding technique

Adhesive bonding technology is widely used in meat recombination due to its excellent bonding properties. The primary binders used in this technique include transglutaminase (TG), glucono-delta-lactone (GDL), fibrin sealant (FS), and gelatin binders. Table 1 presents the processing characteristics and application range of these adhesives.

**Table 1:** Processing characteristics and application range of commonly used adhesives.

Adhesive	Characteristics of Processing	Application
TG	The enzyme exhibits good pH and thermal stability and demonstrates high activity within the range of 45–55 °C and pH 5.0–8.0. The optimum pH is 6.0, and the optimum temperature is approximately 50 °C. Add according to the production requirements.	Ham (Ávila et al., 2014), sausages (Kang et al., 2017), restructured beef (Baugreet et al., 2018)
GDL	Soluble in water (25 °C; 59 g/100 mL), with slow hydrolysis acidification occurring upon heating to about 68 °C; 1% of the aqueous solution has a pH of 3.5, which decreases to 2.5 after 2 h; decomposes at about 153 °C; reduces the generation of nitrosamines; added according to the production needs.	restructured pork (Sadeghi-Mehr et al., 2018)
FS	Reduces the pH value of meat; gel stabilization time of 6 h; at a pH of 6.5, the greatest adhesive strength is achieved, yielding optimal results in preparing restructured meat; the higher the content of magnesium and calcium in the raw material, the better the elasticity and water retention; when the concentration of fibrinogen ranges from 30 to 35 mg/mL, the tensile strength of the prepared gel reaches 160 g/cm^2^, resulting in superior adhesion effects during the preparation of restructured meat; it is mainly used for cold bonding (allowed to stand successively at room temperature of 25 °C and 4 °C).	restructured beef (Liu et al., 2017), restructured pork (Tseng et al., 2006), restructured chicken (Wang et al., 2023)
Gelatin	The pH value ranges from 3.5 to 7.6; when the temperature rises above 40 °C, it coagulates into a transparent elastic gel characterized by its heat-reversible nature.	restructured pork jerky (Kim et al., 2020), sausages (Rosmawati et al., 2023) ham (Lee et al., 2022)

#### 3.1.1 Transglutaminase

**Fig. 2:**
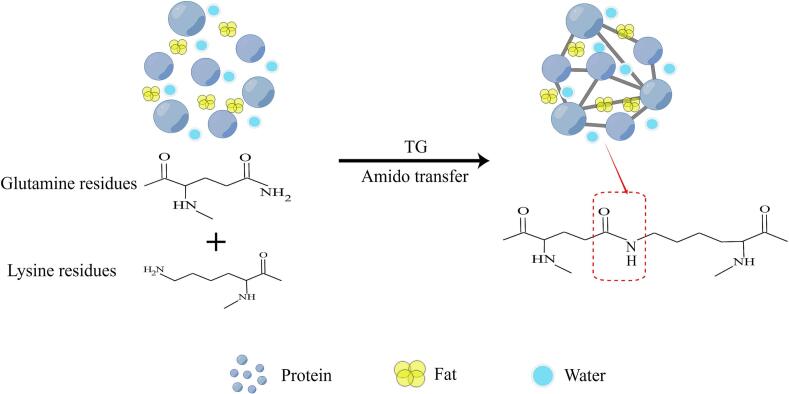
TG catalyzed covalent cross-linking process (By Figdraw).

TG is widely used in the meat industry due to its superior adhesive properties. The industrial production of TG enzyme primarily relies on microbial fermentation (Zhou et al., 2017). TG uses myosin or actin in muscle as a substrate to catalyze the amide transfer reaction between glutamine and lysine residues in the protein. This catalysis leads to the formation of covalent cross-links within and between proteins polypeptide molecules, resulting in aggregation with high viscosity and high molecular weight. Consequently, a dense three-dimensional (3D) network structure is formed, and small pieces of minced meat are bonded to achieve the purpose of recombination (Fig. 2) (Du, Cao, et al., 2022). The restructured meat treated with TG remains intact after freezing, slicing, and cooking.

TG exhibits strong adhesion properties and can be applied to recombine pork to produce new pork sausage products. It catalyzes the transfer of acyl groups and induces covalent cross-links between myosin and casein, thereby enhancing the elasticity of protein food and improving its utilization rate (Fu et al., 2018; Zhou et al., 2017). Additionally, TG combined with other additives can optimize its effect. For instance, TG can improve the quality deterioration of gel products caused by the addition of antioxidant substances. By reducing the formation of disulfide bonds between myofibrillar proteins, TG enhances the hydrophobicity of protein structure, reduces irregular cross-linking of myofibrillar proteins, improves the uneven protein aggregation caused by high-dose antioxidant chlorogenic acid and the formation of irregular macropore gels. This ultimately enhances the water-holding capacity and viscosity of gels (Chang et al., 2023). Since TG is abundantly present in animal tissues and lysine isopeptide bonds (amide bonds) formed by TG catalysis are also found in daily foods, TG is deemed safe for restructured meat processing. Presently, the U.S. Food and Drug Administration (FDA) classifies TG as generally recognized as safe for use in meat products (FDA, 2022).

#### 3.1.2 Glucono-delta-lactone

**Fig. 3:**
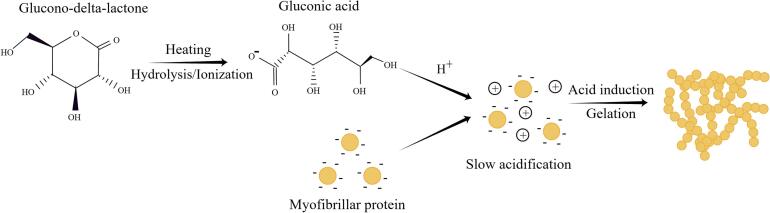
GDL gel adhesion mechanism (By Figdraw).

GDL functions as an acid-type coagulant, incapable of precipitating protein on its own. However, under heating conditions, it hydrolyzes to gluconic acid, which results in a decrease in pH (Xiang et al., 2021). This increase in acidity neutralizes the negative charge of protein molecules, reduces the repulsive force, and facilitates the formation of a dense and uniform gel precipitate (Fig. 3) (Xu et al., 2024; Zhang, 2002). Therefore, restructured meat using gluconolactone requires heating to achieve the desired binding effect. GDL has excellent adhesion and improves gel whiteness and water-holding capacity. The addition of GDL gradually exposes hydrophobic groups inside the surimi protein molecules. With increasing GDL content, the hydrogen and ionic bonds are gradually reduced due to destruction, while the hydrophobic interactions are significantly enhanced, forming a surimi gel network. Optimal strength, hardness, palatability, and viscosity of the surimi gel are achieved when the amount of GDL is added up to 3%. Excessive GDL leads to a continuous decrease in pH and the protein, strengthens the surface charge repulsion of molecules, hinders the formation of protein aggregates, and decreases gel strength (Xiang et al., 2021). Furthermore, acid-induced gel by GDL exhibits higher thermal stability than traditional salt-induced gel under certain conditions. The structure of the salt-induced gel experienced severe damage after heating, with re-established hydrogen bonds between the protein molecules upon cooling but without full recovery gel strength. Notably, the strength and stability of acid-induced gel (adding 1%–4% GDL) surpass those of salt-induced gel when inducing soy protein cold gel (Ni, Fu, et al., 2018). GDL also has emulsifying and preservative properties, which improve the effectiveness of colorants. The joint Food and Agriculture Organization (FAO) and World Health Organization (WHO) expert committee on food additives (JECFA) established an acceptable daily intake (ADI) of 0–50 mg/kg of body weight for GDL in 1998 and subsequently changed it to an ADI “not specified” at its thirtieth meeting (JECFA, 1998).

#### 3.1.3 Fibrin sealant

**Fig. 4:**
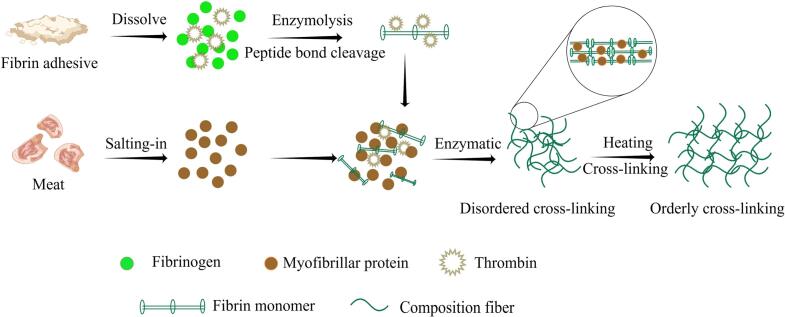
Mechanism of action of FS in meat (By Figdraw).

FS, also known as fibrin glue, functions as a biological adhesive. During the formation of blood clots, fibrinogen in FS is catalyzed by thrombin to convert into fibrin, which forms a network structure that functions as a bonding agent. FS can aggregate into a cold gel at room temperature, and when added to myofibrillar proteins, the FS network is interspersed between the myofibrillar proteins. After heating treatment, myofibrillar protein and FS molecules were denatured and amplified, and the secondary structure of the protein changed from a disordered state to an ordered state. Additionally, the exposure of hydrophobic groups and sulfhydryl groups strengthened the hydrophobicity of the protein surface and the interaction between proteins. This resulted in a dense network structure of the composite gel with excellent texture properties and water-holding capacity (Fig. 4) (Du, Zhou, et al., 2022; Li et al., 2024; Yan, Li, et al., 2024). Several researchers in the field of medicine have explored the application of FS for meat product recombination due to its excellent adhesive properties. Ávila et al. (2014) added FS and thrombin as binders to minced pork. The FS bonding formed a gel that effectively recombined the minced meat. The 3D network structure of the FS was stronger than that of the TG enzyme, which effectively prevents the loss of nutrients and water in restructured meat (Liu et al., 2017). Additionally, combining calcium chloride with FS can produce a synergistic effect, enhancing the binding effect and sensory texture of restructured meat (Tseng et al., 2006). The European Food Safety Authority (EFSA) has assessed FS and concluded that there are no adverse effects from FS originally derived from edible animal parts (EFSA, 2005). Therefore, FS emerges as an excellent option for future research and development of restructured meat.

#### 3.1.4 Gelatin

**Fig. 5:**
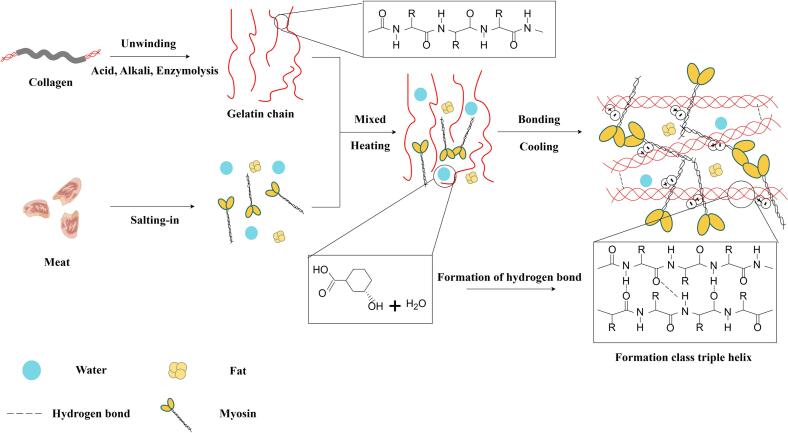
Mechanism of action of gelatin in meat (By Figdraw).

Animal skin or bone collagen is treated with acid or alkali to produce type A and type B gelatin, which can also be prepared enzymatically. Enzymatically hydrolyzed gelatin offers a narrow molecular weight distribution of hydrolysates, exhibiting better binding properties for restructured meat, making it widely used in the food industry (Maki & Annaka, 2020). The stabilized gelatin gel network mainly relies on electrostatic interaction and hydrogen bonding, and its formation is unaffected by disulfide bonds (Haug et al., 2004). As the temperature of the gelatin solution decreases below the transition temperature (35–40 °C), gelatin molecules transition from a random helical conformation to a partially ordered structure of collagen triple helix (Maki & Annaka, 2020). The free hydroxyl group of serine in gelatin combines with water molecules through hydrogen bonds, which increases the gelation viscosity and shear stress in the myofibril mixture (Qiao et al., 2013). Gelatin molecules in restructured meat bind a significant amount of water by forming hydrogen bonds, which impedes the flow of water. At the same time, hydrogen bonding interaction between gelatin molecular chains intensifies to form a stable network structure. Under certain conditions (temperature and pH value), increased electrostatic interaction between myosin in meat and gelatin advances denaturation and gelation, which facilitates bonding (Fig. 5) (Yan, Xie, et al., 2024; Yang et al., 2007). Gelatin can be used in food as a stabilizer, gelling agent, and emulsifying agent. An ADI “not limited” was established for gelatin at the 14th JECFA meeting (1970) (JECFA, 1970).

### 3.2 Emulsifying gel bonding technology

The low productivity and high cost of TG enzyme make it unsuitable for industrialization (Fatima & Khare, 2021), thus leading to the emergence of emulsified gel processing technology. Emulsification involves the combination of myofibrillar protein and fat globules through hydrophobic bonding. This process fills the protein gel network voids with fat, strengthens the protein backbone structure, and reduces the gel pores. Some of the exposed hydrophobic areas of the myofibrillar protein and fat globules combine to form an interfacial protein membrane, which prevents the formation of stable intermuscular and intramuscular fat combinations and increases the bonding of minced meat (Fu et al., 2018; Gravelle & Marangoni, 2021). Emulsion gelation technology in restructured meat production primarily utilizes hydrophilic colloid, phosphate, starch, cellulose, and myofibrillar protein to produce gelation. The application of emulsified gel in restructured meat products is presented in Table 2.

**Table 2:** Application of emulsification gel technology in restructured meat products.

Adhesive	Product	Addition proportion	Effect	Potential challenges
Modified cassava starch (cross-linked and acetylated cassava starch) (Wei et al., 2023)	Pork ball (pork)	4%–8%	Reduce cooking loss and improve water retention, emulsion stability, viscosity, and elasticity.	Acetylated cassava starch may exert a certain pressing effect on the protein gel network.
Phosphate, potassium chloride, and cassava starch (Cruz-Romero et al., 2022)	Sausage (pork)	0.25% phosphate	Increases water holding capacity, hardness, and adhesion; reduces cohesion.	Texture may be tougher compared to sausages made with sodium chloride alone.
Phosphate (tetrasodium pyrophosphate and sodium tripolyphosphate) (Glorieux et al., 2017)	Sausage (pork)	0.06%	Improves emulsion stability and reduces cooking loss.	Acidic sodium pyrophosphate decreases pH and water-holding capacity; Disodium phosphate increases cooking loss and reduces emulsion stability.
Basil seed gum (Lee & Chin, 2020)	Sausage (pork)	1%	Increases hardness and decreases elasticity.	Salt concentration and basil seed gum affect both hydrophobicity and sulfhydryl content, leading to decreased sausage hardness and elasticity.
Kappa carrageenan (Cao et al., 2022)	Sausage (pork)	0.1%–0.4%	Increases water holding capacity and stiffness, reduces cooking loss, and improves emulsion stability.	Excessive kappa carrageenan reduces elasticity.
Iodocarrageenan and sodium tripolyphosphate (Schutte et al., 2021)	Ham (ostrich)	0.2%, 0.35%	Reduces cooking loss; increases hardness, cohesiveness, and gum content.	Ham prepared with sodium tripolyphosphate has the lightest color and the least red color.
Locust bean gum and xanthan gum (Chattong et al., 2015)	Minced meat (ostrich)	1%	The addition of locust bean gum has higher elasticity compared to that of xanthan gum.	Meat emulsion formed by a single or a small quantity of xanthan gum and carboxymethyl cellulose may be less elastic.
Cellulose (MC, CMC, and MCC) (Shao et al., 2020)	Meat batter (beef)	2%	MCC improves the adhesion and texture of low-fat meat batter.	CMC may be detrimental to the network structure of the meat batter.

#### 3.2.1 Hydrophilic colloid

The hydrophilic colloid is a polysaccharide with a high molecular weight that is extracted from plants or synthesized by microorganisms or by chemical action. Its molecules contain a large number of hydrogen bonds, which facilitates combination with water. High water absorption enhances food taste and nutritional value (Li & Nie, 2016). Alginates that are commonly used as hydrophilic colloidal adhesives in production are also known as alginate glue. Among them, potassium alginate enhances gel strength, elasticity, and product texture (Shi et al., 2021). Alginate exhibits excellent adhesive properties and achieves structural stability by cross-linking reactions with Ca^2+^ and Zn^2+^ ions at room temperature to enable efficient bonding of the minced meat (Liu et al., 2013). Furthermore, alginate decomposes in the body to produce glucuronic acid, which inhibits pancreatic lipase activity and reduces the digestion and absorption of dietary triacylglycerol for dietary calorie management (Wilcox et al., 2014). It also has the potential to reduce certain cardiovascular risks (Frey et al., 2014). Besides alginates, hydrocolloids widely used in restructured meat products include biopolymers such as xanthan gum, galactomannans such as Konjac glue and guar gum (Hamid & Navaranjan, 2023), as well as carrageenan and sodium caseinate.

The adhesive properties of hydrophilic colloids are selective and vary across different products. For instance, Wang et al. (2016) found that hydrophilic colloids filled in the protein of *hypophthalmichthys molitrix* made the pores smaller and improved the gel network structure and strength to a certain extent. However, the high gel properties of *Nemipterus virgatus* make it challenging to add hydrophilic colloids into the network structure of surimi protein, resulting in the aggregation of colloidal blocks between proteins. This aggregation may hinder the cross-linking of surimi protein and the formation of continuous network structure, thereby reducing the gel strength, hardness, cohesiveness, and chewiness of *Nemipterus virgatus* surimi products. Additionally, due to the diverse nature and characteristics of colloids, as well as different sources and types, hydrophilic colloids are more likely to take advantage of the synergistic effect of compounding (Fan et al., 2023; Ni, Cao, et al., 2018). For instance, the fragments without branched chains on locust bean gum can form stable connections with helical structures (such as xanthan gum) or double-helical hydrophilic colloids (such as carrageenan) at room temperature. Carrageenan, known for its brittle nature and low elasticity at low pH level, can be combined with TG enzyme to improve the hardness of meat paste, enhance viscoelasticity, and mitigate the brittleness associated with the use of carrageenan alone (Yun et al., 2024). The maximum use of carrageenan should be in accordance with GMP in the General Code of Food Additives standard CODEX STAN 192–1995 issued by the Codex Alimentarius Commission (CAC).

#### 3.2.2 Phosphate

Phosphate is a commonly used water-retaining agent in meat processing. Its main function is to increase the dissolution of myosin by increasing the ionic strength while also enhancing the emulsification gel effect and retaining water. Sodium pyrophosphate, sodium tripolyphosphate, and sodium hexametaphosphate are commonly used in the processing of meat products (Wu, Zang, et al., 2023). Sodium pyrophosphate facilitates actomyosin dissociation and, when combined with tumbling, improves muscle tenderness. It has a certain metal ion chelation, which significantly inhibits the oxidation of fat in meat (Lu et al., 2021). The effect of sodium tripolyphosphate on meat products can be correlated with the quantity added. A small quantity of sodium tripolyphosphate (≤ 0.3 g/100 g) promoted the phosphorylation of proteins in surimi or crab meat. Under the action of static electricity, the phosphorylated protein produces a good protein-tangled network. This network increases the breaking force, water-holding capacity, water distribution of the gel, reduces cooking loss, and improves the water-holding capacity and texture sensory characteristics. However, a higher quantity of sodium tripolyphosphate inhibits protein phosphorylation, resulting in a decrease in the content of α-helix in the secondary structure of mixed gel protein, thereby reducing the effect of gel and adhesion (Zhu, Nie, et al., 2022). Furthermore, sodium hexametaphosphate can affect water retention in restructured meat by chelating metal ions. This influences the activity of the enzyme triphosphatase, which in turn affects the swelling efficiency of myofibrillar fibrillar proteins and the water-retaining effect of other phosphates (sodium tripolyphosphate and sodium pyrophosphate). Sodium hexametaphosphate can be used in meat products such as sausages and hams to improve gel strength, water-holding capacity, and adhesiveness while preventing fat oxidation by metal ion chelating effect (Hu et al., 2021). The maximum use of phosphate in processed meat, poultry and game products is 2200 mg / kg in the general Code of Food Additives CODEX STAN 192–1995 issued by the Codex Alimentarius Commission (CAC).

#### 3.2.3 Starch

Starch can be categorized into amylose and amylopectin based on its molecular structure. Both components contribute to improving the bonding strength between meat blocks, optimizing the organizational structure of meat products, and promoting the improvement of yield. Amylose has poor water solubility, is insoluble in fat, and forms gel upon cooling. On the other hand, amylopectin is insoluble in cold water, soluble in hot water, sticky, and does not gel after cooling. Amylopectin has superior adhesion and stability compared to amylose. When heated in water, starch undergoes gelatinization. As the temperature of gelatinized starch molecules decreases, amylose molecules are entangled with each other and tend to align, facilitating the formation of hydrogen bonds between chains. Protein molecules in meat are then combined with this structure through hydrogen bonds (Xu et al., 2021), resulting in a starch and protein double gel structure. Amylopectin, with its more branched network structure and stronger particle swelling ability, forms a highly branched and dense 3D network structure with protein, which has superior gel properties (Zhang et al., 2023). During the rapid cooling of gelatinized starch to room temperature, the hydrogen bonds between starch molecular chains are mutually constrained and restricted, leading to smaller free space, destruction of the gel structure, and aging. Amylose is more prone to aging than amylopectin. The aging of starch in restructured meat leads to texture hardening, poor taste, and reduced adhesion. Commonly used starches in the processing of meat products include cassava starch, corn starch, and wheat starch. However, due to the limitations of natural starch in water solubility, thermal viscosity, and stability, it is gradually being replaced by modified starch in production. Modified starch, obtained through physical, chemical, or enzymatic treatment of natural starch, changes the original starch molecular size and particle properties or introduces new functional groups. This modified starch interacts with reactive groups in myofibrillar protein, such as –NH_2_, –OH, and –SH, participates in the cross-linking reaction within the protein, and interacts with other biological molecular groups to further improve the gel properties and adhesive effect (Wu et al., 2020). Modified starch used in meat processing mainly includes acetylated starch, esterified starch, oxidized starch, and cross-linked starch.

#### 3.2.4 Cellulose

Cellulose has several hydroxyl groups, which form a strong gel network structure through hydrogen bonds. Cellulose gels based on their derivatives can be in the following forms: methyl cellulose (MC), hydroxypropyl cellulose (HPC), hydroxypropyl methyl cellulose (HPMC), carboxymethyl cellulose (CMC), and microcrystalline cellulose (MCC). These cellulose gels utilize their high aspect ratio to establish a strong bonding network with hydrogen bonding interactions between actin, myosin, and collagen. This enhances the protein network structure between fat and meat particles, increases the viscosity between restructured meat components, maintains the stability of the product, and ensures strong bonding (Gibis et al., 2015; Lee et al., 2023). Although cellulose has adhesiveness, its bonding strength is relatively weak. It is best used in combination with other adhesives (Ahmad et al., 2020). Cellulose maximum usage is in accordance with GMP in the Codex Alimentarius Commission (CAC) food additives standard CODEX STAN 192–1995.

### 3.3 Physical processing bonding technology

**Fig. 6:**
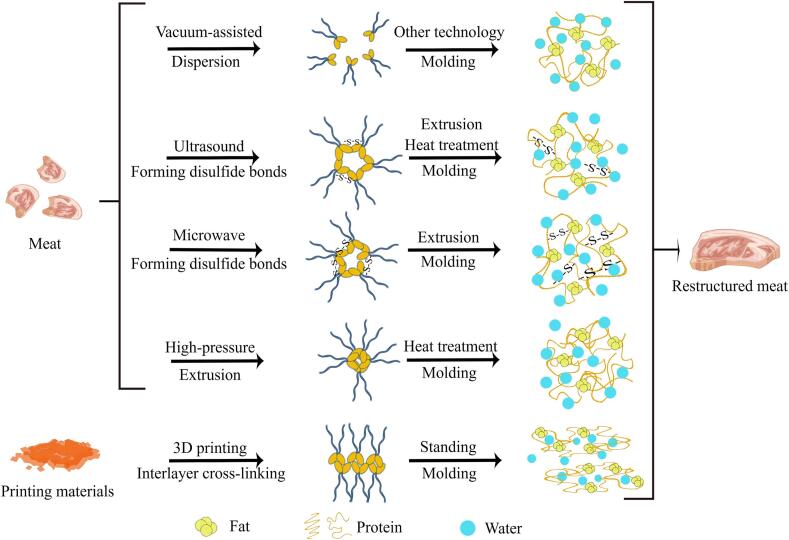
Bonding mechanism of physical processing technology (By Figdraw).

Mechanical heating or pressurization are commonly employed techniques in physical processing. Through mechanical action, meat can be recombined into complete products by forming gel bonds with its myofibrillar protein, thereby improving processing efficiency (Yang et al., 2023). In addition to enhancing adhesion physical processing offers other benefits, such as effectively killing bacteria in restructured meat through high pressure and ultrasound, extending product shelf life, and improving meat tenderness (Zhou et al., 2022; Zou et al., 2023). The main drawback of physical processing technology is that, compared to using adhesives for restructuring, the initial purchase and subsequent maintenance costs of the equipment are higher. Among these techniques, restructuring meat utilizes the primary physical processing bonding technology, as outlined in Table 3, along with the mechanism illustrated in Fig. 6.

**Table 3:** Physical processing adhesion technology in restructured meat.

Physical processing bonding technology	Effect
High-pressure treatment (Zou et al., 2023)	Under normal or low-temperature conditions, high pressure induces the unfolding and dissolution of myofibrillar protein, leading to gelatinization. Under heat treatment, myosin-heavy chains can cross-link and aggregate, forming a denser and more stable gel structure through disulfide bonds and hydrophobic interactions.
Ultrasonic treatment (Zhou et al., 2020)	It provides additional energy to active sulfhydryl groups, elevating them to a high-energy state and reducing the activation energy required for their conversion into disulfide bonds. During extrusion or heat-induced gel formation, protein molecular chains become closely aligned, enabling high-energy active sulfhydryl groups to transform into disulfide bonds between protein chains, enhancing the cross-linking force of the myosin gel network and achieving adhesive effects.
Vacuum-assisted (Zhou et al., 2022)	This technique weakens the binding effect of protein molecules, reduces chemical bonds, extends the molecular structure of proteins, promotes the dissociation of actomyosin and tropomyosin, and facilitates gel network formation.
Microwave processing (Liu & Lanier, 2016; Ye et al., 2021)	Prevents protein molecule degradation and promotes their aggregation into larger particles, thereby promoting protein–protein interactions and generating more disulfide bonds. Cross-linking of proteins with higher molecular weight eventually leads to a denser 3D microstructure, increased gel strength, and improved functional and mechanical properties of the gel.
3D food printing technology (Park et al., 2023)	This technology utilizes meat and fat paste to naturally connect muscle and fat through a single nozzle, printing them in fiber form. Myosin contributes to forming a 3D viscoelastic gel matrix through hydrophilic–hydrophobic balance and fiber structure. Actin and myosin bind minced meat, while TG catalyzes the formation of peptide bonds between glutamine residues and lysine residues in gelatin, stabilizing the structure and integrity of fibers and enhancing the stability of restructured meat.

## 4 Application of bonding technology in various types of restructured meat products

Various recombination techniques use different adhesives or processing methods, each with distinct principles and effects. In meat products, when the matrix protein content is high, it facilitates a more effective combination with binders, enabling the molding of meat re-bonding, thus facilitating the recombination of meat particles or meat. However, stronger adhesives are required to maintain the stability of meat with high fat content. Additionally, the combination of different bonding techniques and other processes often produces synergistic effects.

### 4.1 Restructured pork products

Pork fibers are relatively thin and soft, with less connective tissue, and have fat and protein content of 4% and 21.8%, respectively. Compared to beef and fish myofibrillar protein gels, pork exhibits a denser network structure and higher gel strength. Consequently, additional adhesives may be needed to maintain the structure during the recombination process. For example, alginate and TG are widely used in pork recombination to enhance the gel network structure (Boles, 2011; Wang et al., 2022; Wood, 2017). TG serves as a binder in cold-preserved reconstituted pork chops. The addition of an appropriate amount of TG enhances hardness and chewiness and reduces the water loss rate of the product. This shows TG's robust protein cross-linking ability (Fu et al., 2018). Moreover, the adhesion of TG has little effect on the flavor of recombinant pork products, thus preserving the meat's original taste and facilitating flavoring. In the case of alginate recombinant pork, the addition of Kappa carrageenan after enzymatic hydrolysis (using bromelain) not only played a bonding role but also significantly improved the shear force, hardness, cohesiveness, elasticity, chewiness, gumminess, and protein digestibility, effectively improving the product quality (Nachomkamon et al., 2022).

### 4.2 Restructured beef products

Beef possesses a tighter texture, higher intramuscular fat content, thicker muscle fiber, and fat and protein content of 4.3% and 22.5%, respectively. In comparison to fish myofibrillar protein gels, beef exhibits a more compact gel structure and greater strength, necessitating stronger adhesive to simulate its natural texture and taste. TG plays a crucial role in promoting protein cross-linking, thus enhancing the stability of meat products (Wang et al., 2022; Wood, 2017). Combining TG with sodium caseinate and sodium alginate could significantly improve the quality of recombinant steak. When 0.32% of TG, 0.40% of sodium caseinate, and 0.31% of sodium alginate are added, sodium caseinate enhances the thermal stability of meat products, protects hydrocolloids during heating, improves the temperature resistance of the adhesive system, and promotes the best effect of TG. This combination improves overall quality by 74.03% compared to the control group (Yang et al., 2020). While ensuring the adhesion effect, using TG and plant components such as pea protein isolate, rice protein, and lentil powder as compound adhesives enhance meat binding and improves the nutritional level of restructured meat. The addition of pea protein isolate and lentil powder to recombinant beef improves water retention, bonding strength, elasticity, and chewiness, reduces cooking loss rate, and increases protein content to 28% (Baugreet et al., 2018). Additionally, plant ingredients provide a unique aroma to the product. Li (2019) studied the effects of different additions of soy protein isolate, starch, and salt on the quality characteristics of ready-to-eat recombinant beef products, determining that the optimum addition of soy protein isolate should not exceed 2%. This addition improved product yield, bond strength, and texture and introduced a pleasant bean flavor.

### 4.3 Restructured chicken products

Chicken muscle fibers are thinner, with a lower fat content of 2.1% and protein content of higher 22.3%. Compared to pork, beef, and fish myofibrillar protein gels, chicken exhibits a larger and irregular cavity structure with a rough gel structure. Consequently, less adhesive is required during recombination. This characteristic makes it easier to form a delicate texture during recombination. For example, the use of carrageenan improves chicken texture, thermal stability, and rheological properties (Nan et al., 2022; Polášek et al., 2021; Wood, 2017). Cold bonding reduces protein denaturation, improves product texture, and achieves a better bonding effect. For instance, TG serves as an excellent bonding agent in reconstituted chicken, enhancing the elasticity and taste of meat products while preventing cracking when using traditional adhesives such as carrageenan (Zhou, 2018). Varying proportions of TG can improve the quality traits of whole and recombinant chicken. TG-treated recombinant chicken exhibits lower cooking loss and greater tenderness than the control group, with no difference in sensory attributes compared to the whole meat (Kaić et al., 2021). Furthermore, physical processing bonding technology-assisted adhesives enhance product quality. Ma et al. (2016) found that combining ultra-high-pressure technology with carrageenan addition strengthens the gel effect of chicken breast minced meat, improves taste, enhances water retention, and reduces cooking loss. Under pressure, changes in non-covalent bonds (hydrogen, ionic, and hydrophobic bonds) alter protein tertiary and quaternary structures, making the protein structure stretch and become loose, thereby enhancing solubility and gel properties. Similarly, Wang et al. (2023) used ultrasound recombined chicken, making the structure of myofibrillar and plasma protein tend to be unfolded and easier to connect with each other. Adding an appropriate amount of FS forms a more stable 3D spatial network structure, increasing the viscosity, water retention, elasticity, and hardness of recombinant chicken.

### 4.4 Restructured fish products

Fish meat differs from livestock and poultry meat in that it has shorter muscle fibers, looser tissue structure, and higher water content. With fat and protein contents ranging from 0.2% to 25% and 18% to 20%, respectively, fish meat exhibits lower gel strength, a looser network structure, and poorer water retention. Therefore, water retention agents like phosphate and soy protein may be necessary during the recombination process (Kaić et al., 2021; Wang et al., 2022). Low adhesion recombination improves resource efficiency and sustainability in fish processing (Tokay et al., 2022). For example, in traditional surimi processing, water-soluble fish protein is lost due to multiple rinsing. Ye et al. (2023) used recovered frozen fish protein to promote the cross-linking between recombinant fish protein via TG treatment, resulting in fibrotic surimi with good water-holding capacity, whiteness, and texture characteristics. The addition of soybean protein to low-value fish surimi improved commercial value, gelation, water holding capacity, and overall product quality. Chen et al. (2018) catalyzed covalent cross-linking reactions between soy protein isolate and fish protein using TG, producing super-large protein molecules that significantly improved the gelation and water retention of low-value fish recombinant steaks. Additionally, the adhesion effect of TG in fish can be improved by physical methods. Meng et al. (2020) employed ultrasound-assisted TG to recombine grass carp meat, enhancing hydrophobicity and aggregation between or within protein molecules. Both ultrasound and TG promoted the cross-linking of grass carp meat proteins, resulting in improved cross-linking effects when used simultaneously.

### 4.5 Other restructured meat products

Dry-cured hams are popular among consumers globally. Bone-in ham is rich in flavor and tight and chewy texture, but they have long production cycles and are inconvenient to eat. However, boneless recombinant ham has a shorter processing cycle and is convenient to eat, but the tissue structure and flavor are not as good as that of bone-in ham. The interaction between TG and compound salt enhances tissue stability, adhesion, and flavor in reconstituted ham. When composite salt and TG were used to bond and process boneless pig hind leg meat, observations revealed that the mechanical properties of recombinant air-dried ham became more stable with increased air-drying time, with adhesion force gradually reaching the maximum. Scanning electron microscopy showed that the degree of protein cross-linking was the best (Ávila et al., 2014). However, the color and flavor of this recombinant ham did not meet expectations. Thus, Fulladosa et al. (2009) explored the synergistic effect of high-pressure technology and TG to produce recombinant dry-cured ham. This resulted in a redder, brighter ham with increased color stability and significantly improved flavor (umami and sweet tastes) but with reduced water-holding capacity and elasticity.

Excessive daily sodium salt intake poses health risks, prompting research into sodium salt substitutes for dietary salt reduction. However, different sodium salt substitutes not only affect flavor but also affect the texture of restructured meat. Barretto et al. (2020) demonstrated that adding potassium chloride with ultrasonic treatment to restructured cooked ham can partially replace sodium chloride, enhancing salty taste, myofibrillar protein solubility, water binding force, hardness, chewiness, and flavor. In another study, Pietrasik and Gaudette (2014) replaced sodium chloride with natural low-sodium sea salt in the restructured cooked ham formula. While water retention, texture, and shelf life remained unaffected, consumer evaluations noted a reduction in flavor and aftertaste.

In addition to common pork restructured ham, studies have explored the use of duck and fish to produce restructured ham. For example, Kim et al. (2018) used hydrophilic colloids (alginate, konjac, and carrageenan) to recombine duck skin and meat into duck ham. The hardness, cohesion, gumminess, and chewiness of duck ham were optimized by adding 1% alginate or 0.5% alginate, while 0.5% konjac was added to improve the quality. Thereinto, konjac possesses strong water-binding capabilities, which can synergistically enhance gelation and water retention in minced meat products. Similarly, Alves et al. (2021) used carrageenan and TG to restructure fish into cooked ham, resulting in reduced cooking loss and improved hardness, cohesiveness, chewiness, and tensile strength. The application of bonding technology in meat products is summarized in Table 4.

**Table 4:** Study on the application of common restructured meat adhesives.

Research object	Adhesive	Auxiliary process	Effect
Pork products	Kappa carrageenan (Chen et al., 2023)	N/A	Improve water holding capacity and gel strength
	Carrageenan and TG (Li et al., 2023)	Ultrasonic treatment	Reduce cooking loss, improve water holding capacity, and gel strength
	Kappa carrageenan (Nachomkamon et al., 2022)	N/A	Improve shear force, hardness, cohesiveness, elasticity, chewiness, and gumminess
	TG, calcium alginate, and GDL (Sadeghi-Mehr et al., 2018)	N/A	Improve elasticity and viscoelasticity
	TG and phosphate (sodium tripolyphosphate) (Lesiow et al., 2017)	N/A	Reduce cooking loss, increase hardness, and improve the rheological properties of pork weak gel
	GDL and Kappa carrageenan (Chun et al., 2014)	Pressure	Reduce hardness and increase viscosity
	Konjac glue (Kim et al., 2019)	N/A	Improve water holding capacity, cooking loss, emulsion stability, texture properties, and viscosity
	Carrageenan (Kim et al., 2021)	Super-heated steam drying	Improve tenderness, juiciness, and overall acceptability scores
	Sodium caseinate, Carrageenan, and Konjac glue (Zhu et al., 2019)	Pressure transformation rolling	Improve tenderness, water retention, and reduce cooking loss
	Compound phosphate and TG (Fu et al., 2018)	N/A	Increase water holding capacity, hardness, and chewiness
	Gelatin and Carrageenan (Kim et al., 2020)	N/A	Significantly higher pH value, improved processing yield, and rehydration ability
	Gelatin and TG (Lee et al., 2022)	N/A	Improve water holding capacity
Beef products	Konjac glue, Kappa carrageenan, and Tragacanth gum (Atashkar et al., 2018)	N/A	Konjac glue increased hardness and gel stability, kappa carrageenan improved chewiness, and tragacanth gum increased elasticity
	Konjac powder, Kappa carrageenan, and red yeast rice extract (Widjanarko et al., 2018)	N/A	Enhance water holding capacity and elasticity; the breaking strength of the compound gel is four times that of kappa carrageenan gel
	TG and bacterial concentrate (Merenkova et al., 2019)	N/A	Increased elasticity
	TG, sodium caseinate, and sodium alginate (Yang et al., 2020)	N/A	Improve tenderness, gel strength, and water-holding capacity
Chicken products	TG (Kaić et al., 2021)	N/A	Reduce cooking loss and improve gel strength
	Carrageenan (Ma et al., 2016)	Extra-high voltage	Strengthen the gel effect, improve taste, enhance water retention, and reduce cooking loss
	Phosphate (Garner et al., 2020)	N/A	Improve water holding capacity and gelation
	TG and Casein (Zhou, 2018)	N/A	Improve cohesiveness
	FS (Wang et al., 2023)	Ultrasonic treatment	Improve cohesiveness, hardness, elasticity, and water retention
	FS (Du, Zhou, et al., 2022)	N/A	Improve texture, viscoelasticity, and water-holding capacity
Fish products	Sodium caseinate (Lv et al., 2023)	N/A	Enhance whiteness, water-holding capacity, gel strength, and texture
	TG (Vácha et al., 2006)	N/A	Improve texture and water retention capacity
	TG (Ye et al., 2023)	N/A	Good water holding capacity, whiteness, and texture properties
	TG (Cao et al., 2018)	Microwave radiation	Improve gel strength and structure is more compact
	TG (Meng et al., 2020)	Ultrasonic treatment	Increase hydrophobicity and gelling
	TG (Chen et al., 2018)	N/A	Improve gelling and water retention
	TG (Yerlikaya et al., 2017)	N/A	Improve strength structure and prolong shelf life
Pork and fish meat	TG (Li et al., 2018)	N/A	Improve gel strength
Surimi and air-dried chicken breast	TG (Zhu, Feng, et al., 2022)	N/A	Thermal stability of actin is better, and the hydrophobic interaction and disulfide bond support high thermal degradation resistance

### 4.6 Adhesive effect between different meats

Due to the difference in meat quality, the effectiveness of adhesives differs. For example, TG application yields varying water-holding capacities across different meats. Restructured pork initially experiences a decrease followed by a slow increase, while restructured fish and beef demonstrate gradual increases, peaking with TG increase (Chen et al., 2018; Fu et al., 2018; Yang et al., 2020). However, in restructured chicken, water holding capacity increases with the increase in TG (Kaić et al., 2021). In terms of shear force, restructured beef decreases significantly with the increase in TG. However, the shear force of restructured chicken juice increased with an increase in TG (Kaić et al., 2021; Yang et al., 2020). Additionally, in terms of cohesiveness, restructured chicken increased significantly, then became smaller and tended to be gentle, whereas the cohesiveness of restructured beef increased with an increase in TG (Yang et al., 2020; Zhou, 2018). Finally, in terms of gel strength, restructured fish, chicken, beef, and pork gradually increase with an increase in TG content (Chen et al., 2018; Jiang et al., 2019; Tseng et al., 2000; Yang et al., 2020).

## 5 Quality and safety of restructured meat

Currently, restructured meat products are primarily processed by adding adhesives and some excipients derived from meat processing by-products. These products enhance the meat's flavor to a certain extent, increase meat utilization rates, improve economic value, and diversify food options. However, safety concerns may arise, such as adulteration of raw materials, improper use of additives, and microbial contamination. Mixing meat from different species makes it difficult to detect adulteration with the naked eye, leading to safety incidents such as the European Union (EU) horse meat scandal in 2013 and the adulteration of “beef and mutton” in the market (Premanandh, 2013). Various meat component detection techniques have been developed to address meat adulteration, including real-time fluorescence quantitative polymerase chain reaction (Zhu, Feng, et al., 2022), deoxyribonucleic acid barcoding (Wang et al., 2021), electronic nose and tongue technology, infrared spectroscopy (Cruz-Tirado et al., 2024), and Raman spectroscopy (Chen et al., 2019).

Most adhesives used in reconstituted meat serve as processing aids, stabilizers, and coagulants, which are added based on production needs with high safety standards. Attention should be given to the use of adhesives to maintain or enhance food quality, stability, and nutritional value while adhering to corresponding usage specifications. For example, the widely used TG adhesive is added as a processing aid in food production. During addition and use, it is crucial to assess the necessity of the process and minimize usage while achieving the desired outcome. TG should be removed before the final product formation, or if the residue remains, it should be minimized to ensure they are harmless and functionally beneficial in the final food product. The production process of meat products is susceptible to microbial contamination, especially in restructured meat, which has a more complex process than traditional meat products. Therefore, stringent hygiene measures are essential. Preservatives and sterilization processes should be selected carefully, and the initial sanitary condition of raw materials should be strictly controlled. The auxiliary materials should be added after sterilization to strengthen the disinfection of the environment and equipment in the production process and reduce microbial pollution in the early stage of processing. After processing cold chain products such as fat cattle and restructured steaks, they should be promptly stored, transported, and sold under cold chain conditions. Restructured meat, made from minced meat, undergoes specialized processing techniques, making it susceptible to contamination by pathogenic bacteria like botulinum toxin, *Staphylococcus aureus*, and spoilage bacteria such as lactic acid bacteria (Li et al., 2020). Botox can produce botulinum toxin in an anaerobic environment, *Staphylococcus aureus* belongs to facultative anaerobic bacteria, and lactic acid bacteria can grow in aerobic and anaerobic environments. Therefore, it is necessary to add antibacterial agents against anaerobic and facultative anaerobic bacteria during restructured meat processing. To minimize the use of chemical preservatives, natural preservatives like nisin can be used in combination to inhibit bacterial contamination, including *Listeria monocytogenes* and *Staphylococcus aureus*, in recombinant meat (Soriano et al., 2004; Wu, Dong, et al., 2023). Additionally, some ingredients in restructured meat products use spices like star anise, garlic, and cinnamon, which contain antibacterial properties. Reasonable addition of these spices can contribute to preservation. For example, recombinant ham is vulnerable to *Listeria monocytogenes*. A process like normal temperature or low-temperature autoclaving can destroy microbial cell membranes, reducing the number of bacteria by 1 to 2 log CFU/g. The addition of bacteriostatic agents like *Streptococcus lactis* with high-pressure steam sterilization can further reduce the number of microorganisms by over 5 log CFU/g, which can effectively prolong the shelf life of the product (Teixeira et al., 2018).

## 6 Conclusions and prospects

In this paper, the mechanism, classification (adhesive, emulsified gel, physical processing bonding technologies), and applications of recombinant meat bonding technology, along with its safety considerations across various types of recombinant meat (restructured pork, restructured beef, restructured chicken, restructured fish, and other restructured meat) were reviewed. Adhesive recombination technology has demonstrated its capacity to enhance the morphology, texture, and flavor of meat, playing a key role in the processing of restructured meat products. The research and application of bonding technology in China started late. Despite the rapid pace of development, there are still some issues that deserve attention. Firstly, there is inconsistency in the quality of TG adhesives produced in China, and there are few studies on adhesives such as GDL, FS, and gelatin. Secondly, from a process perspective, the current adhesives and recombination techniques do not seamlessly integrate with the product process, and some adhesives may even have adverse effects on product quality. Finally, in terms of nutrition, restructured meat products often contain a high level of sodium salt, posing health risks. Therefore, future studies in meat adhesion technology should focus on high-quality TG adhesives, new adhesives, and compound adhesives. Furthermore, there is a need for synergy between bonding technology and product processes. Research efforts should focus on reducing the sodium content of restructured meat by exploring the use of binders. The development of adhesive recombination technology will provide substantial support for the comprehensive utilization of meat, cost reduction in production, and the innovation of new products.

## CRediT authorship contribution statement

**Zhijie Li:** Investigation, Formal analysis, Data curation. **Zhonghai Hu:** Resources, Project administration, Funding acquisition. **Wenyun Xia:** Methodology, Investigation. **Mi Zhou:** Validation, Methodology. **Zhenjie Pan:** Resources, Investigation. **Jingjun Li:** Resources, Project administration. **Zongyuan Zhen:** Writing – review & editing, Writing – original draft, Supervision, Resources, Project administration, Methodology, Investigation, Funding acquisition, Formal analysis, Conceptualization.

## Declaration of competing interest

The authors declare that they have no known competing financial interests or personal relationships that could have appeared to influence the work reported in this paper.

## Data Availability

No data was used for the research described in the article.
